# Can Neutrophil-to-Lymphocyte ratio predict the response to BCG in high-risk non muscle invasive bladder cancer?

**DOI:** 10.1590/S1677-5538.IBJU.2018.0249

**Published:** 2019-04-01

**Authors:** Marco Racioppi, Luca Di Gianfrancesco, Mauro Ragonese, Giuseppe Palermo, Emilio Sacco, Pier Francesco Bassi

**Affiliations:** 1 Department of Urology, Fondazione Policlinico Universitario “Agostino Gemelli” IRCCS - Università Cattolica del Sacro Cuore di Roma

**Keywords:** Urinary Bladder Neoplasms, Neutrophils, Lymphocytes

## Abstract

**Objectives:**

To evaluate the neutrophil-to-lymphocyte ratio (NLR) as a prognostic factor for response of high risk non muscle invasive bladder cancer (HRNMIBC) treated with BCG therapy.

**Materials and Methods:**

Between March 2010 and February 2014 in a tertiary center 100 consecutive patients with newly diagnosed HRNMIBC were retrospectively analyzed. Patients were divided according to NLR value: 46 patients with NLR value less than 3 (NLR < 3 group), and 54 patients with NLR value more than 3 (NLR ≥ 3 group). At the end of follow-up 52 patients were high grade disease free (BCG-responder group) and 48 patients underwent radical cystectomy for high grade recurrence or progression to muscle invasive disease (BCG non-responder group). The average follow-up was 60 months. Intervention: analysis and correlation of preoperative NLR value with response to BCG in terms of recurrence and progression.

**Results:**

The optimal cut-off for NLR was ≥ 3 according to the receiver operating characteristics analysis (AUC 0.760, 95% CI, 0.669-0.850). Mean NLR value was 3.65 ± 1.16 in BCG non-responder group and 2.61 ± 0.77 in BCG responder group (p = 0.01). NLR correlated with recurrence (r = 0.55, p = 0.01) and progression risk scores (r = 0.49, p = 0.01). In multivariate analysis, NLR (p = 0.02) and EORTC recurrence risk groups (p = 0.01) were associated to the primary endpoint. The log-rank test showed statistically significant difference between NLR < 3 and NLR ≥ 3 curves (p < 0.05).

**Conclusions:**

NLR value preoperatively evaluated could be a useful tool to predict BCG response of HRNMIBC. These results could lead to the development of prospective studies to assess the real prognostic value of NLR in HRNMIBC.

## INTRODUCTION

Worldwide, bladder cancer is the ninth most commonly diagnosed malignancy and the 13^th^ cause of cancer deaths in 2015 (1).

Bladder cancer is the second most frequent genitourinary tumor. At first diagnosis of 75 – 85% patients have a mucosal (stage Ta, Carcinoma in situ) or submucosal neoplasia (stage T1) (2).

The challenge in treating non - muscle - invasive bladder cancer (NMIBC) is to preserve the bladder and its function, accepting the risk of recurrence (up to 78% of cases) and the risk of progression to muscle - invasive disease (up to 45% of cases).

The identification of patients with higher risk of recurrence and progression is mandatory in order to predict oncological outcomes and for optimal tailored therapeutic decision - making.

The gold standard treatment for NMIBC is represented by transurethral resection of bladder tumor (TURBT) and a re - TURBT when indicated (any high grade disease except Cis, any T1, incomplete TURBT or absence of muscle in the specimen), followed by adjuvant intravesical chemo - or immunotherapy.

European Organization of Research and Treatment of Cancer (EORTC) risk tables (3) suggested a stratification of patients in low, medium and high risk of recurrence and progression, and a relative treatment strategy.

The treatment of high-risk patients is based on the induction course of intravesical immunotherapy with BCG (Bacillus Calmette-Guerin) followed by maintenance course for at least one year (4).

Many predictor tools have been analyzed in the context of cancer development and progression. A high neutrophil - to - lymphocyte ratio (NLR) was already consistently associated with locally advanced disease and worse general and cancer-specific survival rates in several solid tumors (5).

A high NLR seems to represent an independent prognostic factor of recurrence and progression of disease in patients with NMIBC (6). NLR may be helpful to better identify patients who would be optimally treated and cured with BCG and patients in whom conservative treatment would probably be ineffective.

The purpose of this study is to evaluate whether preoperative NLR measurement may add useful information for the best disease management and for prediction of response to BCG in high risk non muscle invasive bladder cancer (HR - NMIBC).

## MATERIALS AND METHODS

We retrospectively analyzed 100 consecutive patients treated in our Clinic for a first diagnosis of high - risk NMIBC (according to EORTC and EAU guidelines) between March 2010 and February 2012. We excluded patients with previous history of low risk NMIBC.

All these patients underwent an intravesical BCG schedule consisting of one induction course (6 weekly instillations) followed by one year of monthly maintenance course.

In all patients, the histological specimen documented a pure urothelial cancer with detrusor muscle included in the resection; we did not analyzed patients with other histological variants.

All high grade pTa and pT1 patients underwent reTURBT according EAU guidelines.

For all patients the follow-up was 60 months.

At the end of follow-up 52 patients were high grade disease free (BCG - responder group) and 48 patients underwent radical cystectomy for high grade recurrence or progression to muscle invasive disease) (BCG non - responder group).

Patients were divided according to their NLR value (Table-1): 46 patients with NLR value less than 3 (NLR < 3 group), and 54 patients with NLR value more than 3 (NLR ≥ 3 group).

**Table 1:** Baseline characteristics based on NLR value.

Variables		General	NLR < 3	NLR ≥ 3	p value
N° of patients		100	46	54	
Median age**, years ± SD		67.5 ± 10.7	68.6 ± 10.8	66.7 ± 10.5	> 0.05
**Sex** *, **n° of pts (%)**					**> 0.05**
	Male	87 (87%)	39 (84.8%)	48 (89.6%)	
	Female	13 (13%)	7 (15.2%)	6(10.4%)	
NLR**, value ± SD		3.17 ± 1.12	2.32 ± 0.41	3.90 ± 0.88	0.01
**Pathological stage** ***, **n° of pts (%)**					**> 0.05**
	Ta	11 (12%)	3 (6.5%)	8 (14.8%)	
	T1	73 (73%)	35 (76.1%)	38 (70.4%)	
solitary Cis		16 (15%)	8 (17.4%)	8 (14.8%)	
Concomitant Cis*, n° of pts (%)		25 (25%)	2 (4.3%)	23 (42.6%)	0.01
**No. of tumors** *, **n° of pts (%)**					**0.01**
	1	48 (48%)	35 (76.1%)	13 (24.1%)	
	≥ 2	52 (52%)	11 (23.9%)	41 (75.9%)	
**Tumor size (mm)** *, **n° of pts (%)**					**0.01**
	< 30	78 (78%)	42 (91.3%)	36 (66.7%)	
	≥ 30	22 (22%)	4 (8.7%)	18 (33.3%)	
Recurrence risk score**, value ± SD		5.5 ± 2.3	4.2 ± 1.7	6.5 ± 2.2	0.01
Progression risk score**, value ± SD		12.1 ± 3.9	9.7 ± 2.2	14.2 ± 3.9	0.01
**EORTC recurrence risk** ***, **class (%)**					**0.01**
	1-4	39 (39%)	29 (63.1%)	10 (18.5%)	
	5-9	55 (55%)	17 (36.9%)	38 (70.4%)	
	≥ 10	6 (6%)	0 (0%)	6 (11.1%)	
**EORTC progression risk** ***, **class (%)**					**0.01**
	2-6	5 (5%)	3 (6.5%)	2 (3.7%)	
	7-13	58 (58%)	40 (87%)	18 (33.3%)	
	≥ 14	37 (37%)	3 (6.5%)	34 (63%)	

**SD =** standard deviation; Pts: patients; **NLR =** Neutrophil / Lymphocyte Ratio; **Cis =** Carcinoma In Situ; **EORTC =** European Organization for Research and Treatment of Cancer; **Test =** chi-square*; t-student**, ANOVA***

Three patients developed a solitary or concurrent upper tract urothelial carcinoma (UTUC) disease: two patients (4.3%) in NLR < 3 group and 1 patient (1.8%) in NLR ≥ 3 group; two patients (3.8%) in BCG - responder group and 1 patient (2.1%) in BCG - non responder group.

In all patients, the BCG strain used was Seed RIVM by Medac^®^ (derived from seed 1173 - P2, 2 × 10 to 3 × 10 viable units). The Medac^®^ - BCG powder was re - suspended with 50 mL of 0.9% normal saline and introduced into the bladder via a 10-12 French urethral catheter. Patients were instructed to hold the drug in the bladder for two hours.

Four experienced urologists performed all the diagnostic cystoscopies and all the TURBTs.

All specimens were analyzed by an experienced dedicated uropathologist.

The 2009 TNM classification (7) and the 2004 WHO grading system (8) were used for histologic reports.

Recurrence was defined as the first histologically confirmed high grade NMBIC recurrence; progression was defined as the development of muscle - invasive bladder cancer (MIBC).

Diagnostic cystoscopies, TURBTs and eventual reTURBTs were performed using NBI (Narrow Band Imaging) technology.

All the analyzed patients completed the induction six - weekly BCG instillation schedule (in order to avoid bias related to the intravesical immunotherapy toxicity).

At the end of the induction course all patients underwent endoscopic evaluation, voiding and washing urinary cytology, transurethral resection (TUR) of any suspected area. Random biopsies including prostatic urethra for patients were performed if indicated.

In case of high grade tumor recurrence or progression to muscle invasive disease during follow-up, patients were considered as BCG failure according to EAU guidelines and underwent radical cystectomy. We excluded all cases of BCG-intolerance (9). Patients with low grade low stage BCG - relapsing diseases were considered in the BCG responder group considering that they should not be considered as “BCG failure” according to EAU guidelines (3).

Patients with complete response after induction course underwent BCG maintenance course for at least 1 year and subsequent endoscopic follow-up every 3 months for the first two years and then every 6 months according the EAU guidelines (3).

All the analyzed patients completed the induction six - weekly BCG instillation; no event of therapy discontinuation was reported. We reported 5 cases of therapy discontinuation during the last instillations of the maintenance cycle (due to severe BCG - related complications): 3 patients (6.5%) in NLR < 3 group and 2 patients (3.7%) in NLR ≥ 3 group; 3 patients (5.8%) in BCG - responder group and 2 patient (4.2%) in BCG - non responder group; these patients therefore underwent cystoscopic, cytologic and radiologic evaluations: all of them were high grade disease - free, so they continued regular follow-up.

For each patient, we reported the preoperative hematologic and chemical data, including the total number of white blood cells (WBC), neutrophils (N) and lymphocytes (L). Patients underwent blood sampling the day before the TURBT, in the morning, after at least 6 hours of fasting. We enrolled only patients without hematuria in order to avoid any sort of bias, especially in terms of total blood count. The NLR ratio was calculated by dividing the value of N by the value of L.

The preoperative NLR measurement collected at the first TURBT was the reference value for each patient.

We used a receiver operating characteristic (ROC) curve to determine an appropriate cut - off value.

All patients were classified into two groups according to the NLR. The X2 test was used to verify the significance of the correlation between the NLR and the clinic - pathological characteristics.

Patients with preoperative diagnosis of active infection, hematologic neoplasms or unexplained leukocytosis, presence of other neoplasms, prior systemic chemotherapy were excluded from the study.

The groups were compared according to the following data: age, sex, stage and grade of tumor, size and number of tumors, presence of carcinoma in situ, NLR.

In addition, patients were classified according to the EORTC risk tables (3) for the recurrence risk score (1-4, 5-9, ≥ 10) and for the progression risk score (2-6, 7-13, and ≥ 13). No patient had a recurrence or progression risk score of zero and all patients scored zero regarding “prior recurrence rate” because all of them had newly diagnosed bladder cancer.

The aim of the study was to identify a potential role of NLR as an independent prognostic factor for the response to endovesical BCG therapy.

Categorical variables were summarized using actual counts and percentages; the continuous variables using the mean ± standard deviation.

Parametric and nonparametric variables were evaluated using the t - test and the chi square test, respectively.

Logistic regression was used to determine independent predictors of BCG response.

The X2 distribution was used for categorical data. Pearson's test was used for the correlation analysis. Statistical significance was considered at p < 0.05.

Kaplan Meier curves and log rank test were built in order to evaluate cancer free survival between the two groups.

All patients provided informed written informed consent with guarantees of confidentiality.

The protocol for the research project was approved by the local Ethics Committee and it conformed to the provisions of the Declaration of Helsinki (as revised in Fortaleza, Brazil, October 2013).

## RESULTS

The baseline patient's characteristics are summarized in Table-1. The median age of patients was 67.5 ± 10.7 years.

The mean value of NLR in all patients was 3.17 ± 1.12.

The mean NLR value was 2.61 ± 0.77 in BCG - responder group and of 3.65 ± 1.16 in BCG - non responder group (p value: 0.01), and 2.32 ± 0.41 in NLR<3 group and 3.90 ± 0.88 in NLR ≥3 (p value: 0.01).

After 60 months of follow-up, all patients in BCG-responder group were cancer-free, while all patients in BCG-non responder group underwent radical cystectomy for recurrence of high-grade NMIBC (n = 31) or for progression to muscle-invasive disease (n = 17); in 12 cases (70%) with evidence in the post-operative histopathological specimen of pT2, in 4 cases (25%) with pT3 and in 1 patient (5%) with pT4 disease for involving of prostatic stroma at the level of prostatic urethra.

According to the ROC analysis, the optimal cut-off of NLR was ≥ 3 (area under the curve [AUC] 0.760, 95% CI, 0.669-0.850, sensitivity 80.0%, specificity 72.0%, PPV 74.0%, NPV 78.0%, OR 10.29, RR 3.36, +LR 2.85, p value 0.01).

**Figure 1:**
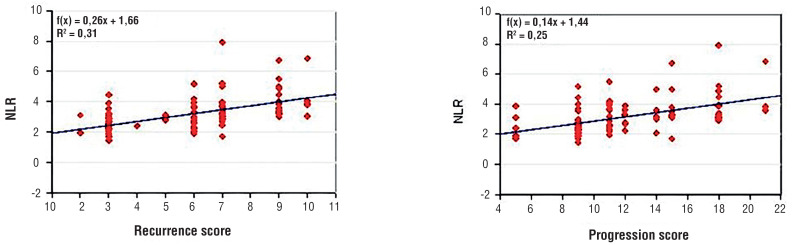
Correlation between NLR and recurrence risk score and progression risk score.

We reported a linear correlation between NLR value and recurrence risk score (r=0.55, p=0.01) and progression risk score (r = 0.49, p = 0.01) considered as continuous variables (Figure-1).

Moreover, we reported a statistically significant difference in the value of NLR among patients with different recurrence (p = 0.01) and progression risk scores (p = 0.01) considered as categorical variables.

Recurrence/progression rates increased with the increase of NLR values: 15.4% in patients with NLR between 1 and 2, 30.3% in patients with NLR between 2 and 3, 62.5% in patients with NLR between 3 and 4, and 78.6% in patients with NLR higher than 4 (p < 0.05).

**Figure 2:**
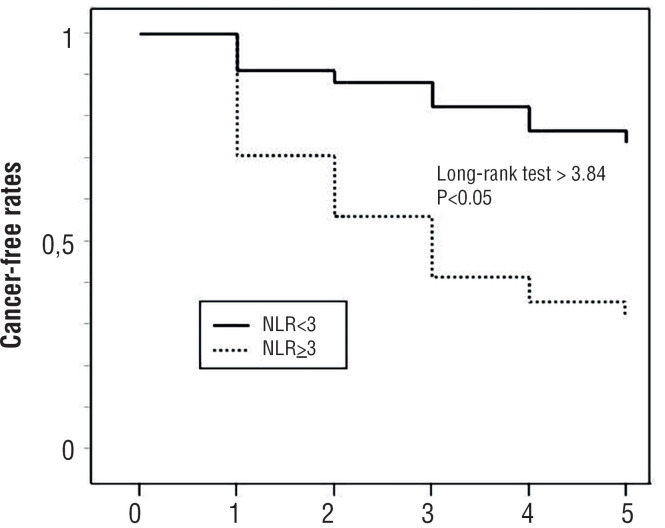
Kaplan-Meier cancer free survival curves for patients with NLR< 3 and NLR ≥ 3.

We built Kaplan-Meier cancer free survival curves for patients with NLR < 3 and NLR ≥ 3: the log-rank test showed a statistically significant difference between the two curves (p < 0.05) (Figure-2).

In the multivariate analysis, prognostic factors were analyzed in order to weigh their role in relation to the primary endpoint; in this analysis we considered NLR and EORTC risk categories according relative scores (1-4, 5-9 and ≥ 10 risk groups for recurrence; 2-6, 7-13, ≥ 14 risk groups for progression): NLR (p = 0.02) and EORTC recurrence risk groups (p = 0.01) were associated, while EORTC progression (p = 0.11) risk groups were not associated (Table-2).

**Table 2:** Logistic regression analysis for response to BCG.

Factors	B	S.E.	t	p	B %95 C.I.	
					Lower	Upper
NLR*	0.077	0.035	2.226	*0.02*	0,008	0,147
EORTC recurrence risk**	0.152	0.048	3.143	*0.01*	0,056	0.248
EORTC progression risk**	0.202	0.122	1.65	*0.10*	-0.041	0.444

* continuous variable;

** categorical variable

At the end of follow-up we reported higher recurrence/progression rates in case of higher NLR values. NLR values increased with the increase in scores: in 1-4, 5-9 and ≥ 10 recurrence risk groups NLR values were 2.60 ± 0.84, 3.44 ± 1.11, and 3.71 ± 0.38, respectively (p value: 0.01); in 2-6, 7-13 and ≥ 14 progression risk groups NLR values were 2.66 ± 0.41, 2.89 ± 1.02 and 3.79 ± 1.26, respectively (p value: 0.01).

At the end of follow-up in BCG- responder group there was no cancer-related death while in BCG-non responder group the cancer specific survival rate was 96.2%; the 2 patients that died presented an average NLR value of 4.8, statistically different compared to disease-free patients (p = 0.01) and patient treated with radical cystectomy (p = 0.01).

## DISCUSSION

The prognostic role of NLR has already been extensively analyzed in many solid tumors but the underlying mechanism explaining the association of a high NLR and poor prognosis / poor outcomes of cancer patients is poorly known (10).

A potential mechanism has been identified in the association between high NLR and inflammation. The systemic inflammatory response stimulated by the neoplasia involves a pro-tumor inflammatory state, leading an active role of systemic inflammation in tumor growth, recurrence, and progression. Many inflammatory indexes (PCR, Platelet/Lymphocyte ratio - PLR -, albumin levels, fibrinogen levels, etc.) obtained from blood tests were associated with outcomes of many cancers.

Neutrophils and lymphocytes have an inhibitory and activating action, respectively, on the immune system: that is why they might reflect the inflammatory and immune response of the host.

Inflammatory response induces neutrophilia, lymphocytopenia and high excretion of proangiogenetic factors, growth factors, anti-apoptotic factors, all stimulating tumor growth and progression (11). Neutrophilia as an inflammatory response inhibits the immune system by suppressing the cytolytic activity of immune cells such as lymphocytes, activated T cells and natural killers cells (12, 13).

The increase in tumor lymphocyte infiltration has been associated with better responses to cytotoxic treatments and better prognosis of cancer patients. Inflammatory cytokines and chemokines can be produced by both tumor and host cells (such as lymphocytes) contributing to tumor progression.

NLR might therefore represent a systemic inflammation parameter and efficient biomarker of the host-tumor interaction (11). A high NLR value could reflect both an increased neutrophil-dependent inflammatory reaction and a diminished lymphocyte-dependent immune response (14).

An increase in NLR was associated with an increase of peritumoral infiltration of macrophages and increased interleukins and cytokines production (IL-1ra, IL6, IL7, IL8, IL9, IL12, IFNgamma, etc.). Neutrophils and other cells secrete tumor growth promoting factors (VEGF, HGF, IL6, IL8, MMPs, elastanes) thus contributing to microenvironment of tumor stimulation (for example, IL6 has been shown to be at higher concentrations in 13 different types of neoplasia and associated with higher tumor stage and adverse prognosis).

In vitro and in vivo studies showed that the systemic and local responses (at the bladder wall) to BCG are represented by an increase in T lymphocytes, with a predominance of T helper / inducer cells.

Although studies demonstrated BCG efficacy (and safety) of immunocompromised patients, studies on the immunological mechanism of BCG therapy showed that an intact immune system (particularly of the cellular system) is required for anti-tumor activity; clinical and laboratory evidence showed that BCG interaction with immune system produces a relative systemic immunity to BCG, necessary for its effectiveness (15).

Previous studies assessed the NLR value in patients with MIBC undergoing radical cystectomy (16): correlations were found between high levels of NLR and diagnosis of MIBC at TURBT and with non-organ confined tumor (17).

In the study of Mano et al. higher NLR values were associated with unfavorable tumor characteristics (high grade of differentiation, T1 tumor) in 122 patients with new NMIBC diagnosis.

In our study, patients with NLR ≥ 3 presented statistically significant worse tumor characteristics (negative prognostic factors) such as concomitant Cis (p<0.01, OR 16.3), multifocality (p<0.01, OR 10), tumor size >3 cm (p<0.01, OR 5.2) compared to patients with NLR <3.

Higher NLR values were associated with tumor progression and recurrence in univariate and multivariate analyses adjusted for EORTC risk groups (16). We observed linear correlations between NLR value and patient's EORTC classes (Figure-1).

In a cohort of 86 patients, Albayrak et al. reported a significant difference in NLR values between recurrence and progression risk score groups, with mean NLR values progressively higher as the risk class increased. In this study, however, patients’ age was statistically different between recurrence and progression risk score groups and, after correcting for the statistical effect of age on scores, the relationship between NLR and recurrence and progression risk scores was no longer significant. Authors suggested to correct the NLR value according to patients’ age in order to avoid deceitful results (18). We evaluated NLR according to EORTC risk score and we reported their linear correlation. Unlike other studies (16, 19), patients’ age was not correlated with NLR values and EORTC risk scores, so our results did not need correction for the patients’ age.

In the study of Cimen et al. in a cohort of 271 patients the NLR value was associated with the T1 pathological stage: patients with NLR >1.8 had 1.5 times higher risk to develop a lamina propria infiltrating tumor. [18] In our study, all patients with NLR<1.8 had a lamina propria infiltrating cancer, so we could not confirm or compare these data, maybe due to the specific subgroup of HRNMIBC patients we considered (Cimen et al. included papillary urothelial neoplasm of low malignant potential – PUNLMP - and low grade low stage bladder cancer patients).

In a cohort of 178 patients, Favilla et al. prospectively assessed the role of NLR as biomarker of NMIBC in terms of prognostic marker of disease recurrence, reporting a statistically significant association of higher NLR value (with a cut-off ≥ 3) with recurrence (such as in our study) but not with progression (differently from our results) (20).

In our study patients with higher NLR showed higher recurrence/progression rates than those with lower NLR: 15.4% of recurrence/progression rate for patients with NLR values between 1 and 2, 32.4% for NLR values between 2 and 3, 83.3% for NLR values between 3 and 4, 85.7% for NLR value more than 4.

Qzyalvachi et al. reported a statistically significant correlation between recurrence of pT1 HGNMIBC and NLR with cut-off of NLR ≥ 2.43 in 166 patients. In the multivariate logistic regression analysis NLR, tumor number and smoking were determined as independent predictors of recurrence while no statistically significant correlation was reported between NLR and progression (18). In our study the multivariate analysis showed that NLR value (p=0.02) and EORTC recurrence (p=0.01) risk groups were independent factors of non-response to BCG (intended as high-grade recurrence or progression disease); even in our study progression risk groups were not independent factors of non-response to BCG (p=0.10)

D’Andrea et al. evaluated the prognostic role of NLR in patients with primitive NMIBC. The optimum cut-off value of NLR was 3. In univariate and multivariate analysis, NLR≥3 was significantly associated with recurrence free survival (RFS) and progression free survival (PFS) and with outcomes in patients treated with BCG. In this retrospective study on 918 patients, authors suggested the integration of NLR into a predictive model to predict RFS and PFS in patients with NMIBC (19).

Mbeutcha et al. showed a relationship between oncological outcomes of NMIBC and markers of systemic inflammatory response, including NLR. Authors evaluated retrospectively 1.117 patients and reported a statistically significant association between high NLR values and disease recurrence and progression; this association was confirmed in the analysis of a subgroup of 300 patients treated with BCG. Even these authors suggested the introduction of NLR in prognostic models (21).

The EAU guidelines for UTUC management already considers NLR as a prognostic tool in the preoperative assessment (22), nevertheless our results and literature suggest a pivotal role for this value in bladder cancer management.

In a retrospective study on 1.551 patients, Kang et al. reported a significant association of high preoperative NLR with host-related outcomes (overall and cancer specific survival) but not with PFS and RFS (23). In this larger series of patients a linear correlation of NLR with RFS and PFS was not reported, but authors reported a significant association between this factor and overall survival and cancer specific survival, still validating the prognostic power of NLR in bladder cancer.

In our cohort of patients the mean higher values of NLR seemed to be compliant with the specific population of patients in exam (high grade, pT1, presence of Cis, etc.). NLR was statistically higher in patients who underwent radical cystectomy; in fact patients with NLR ≥3 showed a 2.85 times higher risk to be treated with radical cystectomy.

Our aim was to add a prognostic factor for the definition of the particular subgroup of patients with “highest risk“ NMIBC (3) who, according to EAU guidelines (LE: 3, Strength rating: Strong), could benefit from radical surgery even after the first diagnosis given the high risk of recurrence and progression.

In addition to the other already known prognostic factors, NLR could help to inform patient, in a shared decision making process, about the aggressiveness of the neoplasm, providing realistic probabilities of success/failure of the conservative treatment.

It is widely known that this group of patients require the best therapeutic option (BCG) and a close follow-up since conservative intravesical treatment has a high probability of failure (up to 62% of recurrence, up to 45% of progression, in our specific cohort of patients, according EORTC class risk).

According to our experience the NLR report could allow to better identify patients for whom radical cystectomy might be the only form of curative treatment. This could also allow to precociously begin a psychological support for the patient in prevision of a major surgery and all related changes on the quality of life (QoL) (24).

Other advantages of using the NLR value are: easy applicability, wide availability and low cost. In the literature, various NLR cutoff values were evaluated and applied (25). This implies the need to interpret the results carefully as the cut-off value was chosen in each specific cohort by testing different discrimination values with relative different sensitivities and specificities. In the light of these considerations, an ideal and generalizable NLR value is still far from being well defined.

Some limitations of our study were: small number of patients; individual preoperative systemic inflammatory response tests; retrospective study performed in a single tertiary center with relative unavoidable selection biases; the lack of in vivo and / or in vitro studies in order to validate our hypotheses.

## CONCLUSIONS

Our data showed that a high value of NLR evaluated preoperatively might be helpful to predict the BCG response and therefore provide critical information for the clinical management of high-risk NMIBC patients together with the prognostic factors already known.

In order to give greater significance to our results, prospective studies are needed for validating the NLR as a real prognostic factor of the high-risk NMIBC and for identifying the ideal and reproducible NLR cut-off value.
